# Metastatic endophthalmitis - Has the trend of causative organism changed in the modern antibiotic era - A Systematic Review

**Published:** 2020

**Authors:** Meenakshi Wadhwani, Sanjay Kumar Mishra, Manika Manika, Shibal Bhartiya

**Affiliations:** * Chacha Nehru Bal Chikitsalya, Geeta Colony, New Delhi, India; ** Army College of Medical Sciences and Base Hospital, Delhi Cantt, India; *** Glaucoma unit Fortis Hospital, Gurugram, India

**Keywords:** metastatic, endophthalmitis, inflammation, infiltration

## Abstract

Endogenous endophthalmitis, EE, a less common form of endophthalmitis, occurs when the microorganisms spread to the eye through the bloodstream, from a septic focus elsewhere in the body, that breaches into the integrity of the eyeball itself. The etiopathogenesis of endogenous endophthalmitis has changed over the past two decades, the aim of this review being to study the changing trends in causative organism in the era of modern antibiotics.

## Introduction

Endophthalmitis is the inflammation of inner coats of the eyeball that progressively involves the vitreous cavity. It is a serious vision threatening complication. For this reason, prompt etiological diagnosis and treatment are imperative in cases of endophthalmitis. Therefore, it is extremely important for the clinician to pick up the early signs and symptoms of the disease, so that the treatment can be initiated immediately, improving final patient outcomes.

Endophthalmitis may be classified as exogenous (post-traumatic or postoperative) or endogenous (metastatic). Exogeneous endophthalmitis occurs when the outer wall of the eye sustains a break due to surgical intervention or trauma or severe infection in cornea or contiguous structures that breach the integrity of globe. 

Endogenous endophthalmitis, EE, is less common and occurs when the microorganisms spread to the eye through the bloodstream, from a septic focus elsewhere in the body. This means that endogenous endophthalmitis is a result of the spread of a blood borne infection, with the primary infective focus being elsewhere, rather than any breaches in the integrity of the eyeball itself. With the advent of effective antimicrobial drugs, endogenous endophthalmitis has become very rare [**1**,**2**]. It usually affects immunocompromised, debilitated and hospitalized patients since they are more susceptible to infections, and instrumentations and intravenous access means they have a higher risk of septicemia and metastatic foci of blood borne infections. Such patients often have signs of sepsis or metastatic infection elsewhere in the body. Though, in today’s scenario, with the advent of modern antibiotic regimens, the occurrence of once common causes of septicemia like Salmonella, Staphylococcus aureus, Escherichia coli, etc. is decreasing; other organisms like coagulase negative Staphylococci, Candida species and non-fermenting gram negative bacilli are causing more and more blood stream infections in immunocompromised, chronically ill and hospitalized patients [**3**-**5**].

This review aims to ascertain if there has been a change in the pattern of ocular manifestations and causative organisms of metastatic endophthalmitis, in the current era of modern antibiotics. 

## Methods

The database search was conducted from January to June 2018. The search engines used included PubMed, Medline, OVID and Google Scholar. The following medical subject heading (MeSH) terms were searched separately and then cross matched: bacterial endogenous or metastatic endophthalmitis, endophthalmitis other than postoperative, while limiting the search to English and human studies. 

From the initial MeSH searches, original articles and review articles that were published after January 2000 were analyzed. An in-depth assessment of articles was carried out; citations, and cross references from relevant key articles were used to identify additional publications.

The inclusion criteria for the studies were: 

• setting: country, inpatients/ outpatients/ both,

• underlying infection: site, organism, susceptibility pattern,

• participants: age and number of participants, outcomes.

The studies with ill-defined visual acuity and not following WHO standard guidelines/ methodology were excluded. Secondary publications reviewing different causes of endogenous or metastatic endophthalmitis were also included. Thus, a total of 37 articles were found to be suitable for inclusion in this review (**Chart 1**).

**Fig. 1:**
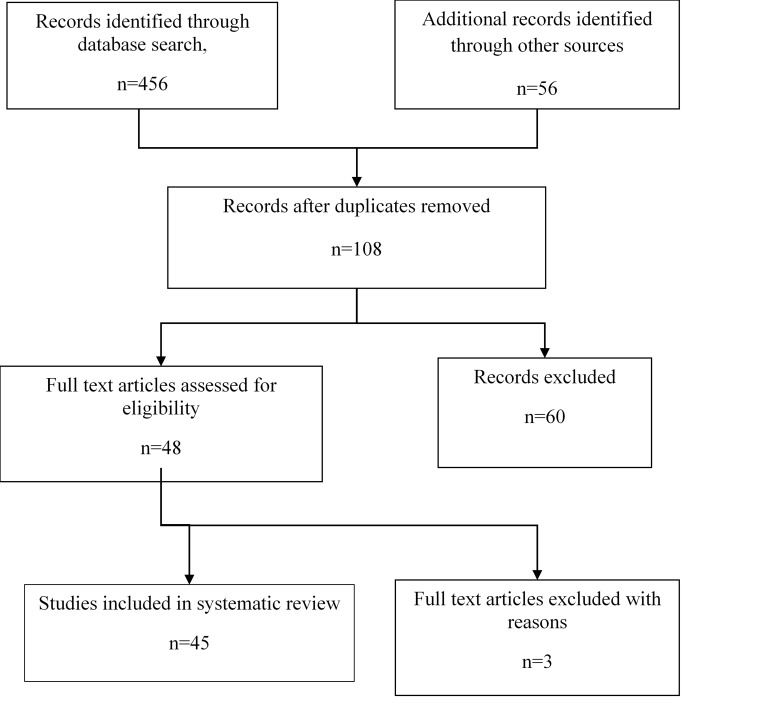
Flowchart depicting summary of review strategy followed for the study

## Results and discussion

As described earlier, EE is a rare entity nowadays because of effective antimicrobial agents and better diagnostic techniques leading to effective treatment of primary site of infection. The etiopathogenesis of endogenous endophthalmitis is briefly described in **Chart 2**.

**Fig. 2:**
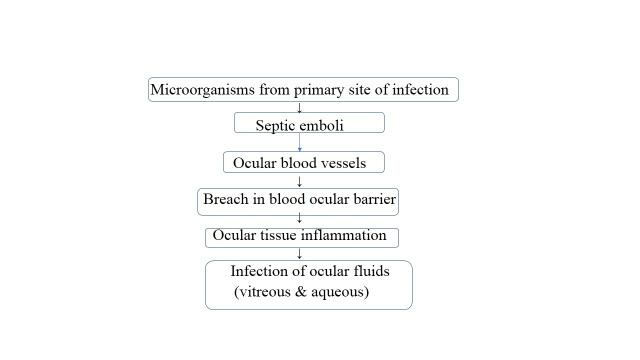
Pathogenesis of endogenous endophthalmitis

EE patients with no obvious primary site of infection should undergo a thorough detailed examination of abdomen, heart, lungs, teeth, limbs, abdomen, that includes investigations like abdominal USG, echocardiography, abdominal/ chest CT, blood/ urine/ sputum cultures.

We analyzed 45 case series and case reports of endogenous endophthalmitis between 2000 and 2018, so as to identify the most common primary sites of infection, most common pathogens and their effective antimicrobial treatment. 

**Liver abscess**

In 2000, Cahil M et al. reported a case of EE associated with liver abscess treated with intravenous Ciprofloxacin and hydrocortisone, topical antibiotic, steroid and mydriatics, PPV+ retinopexy, patient’s visual outcome was PL in R/ E and 6/ 12 in L/ E [**6**]. In 2000, Ang LPK et al. reported a case of EE associated with liver abscess, treated with intravitreal, topical, subconjunctival cefazoline and gentamycin and intravenous ceftriaxone and gentamycin but could not regain any vision [**7**].

In 2003, Tang et al. reported a case of EE associated with suppurative liver disease, the patient was treated with intravenous cefotaxime and intravitreal vancomycin along with amikacin. The outcome of this patient was not encouraging, with a complete loss of vision and the eye ended up in phthisis [**8**]. 

In 2007, Yang et al. reported 22 patients of EE associated with liver abscess, 15 patients were diabetic, biliary stones being present in 2 patients. They were treated with systemic 3rd generation cephalosporins and aminoglycosides. 11 patients had to be eviscerated as the intraocular inflammation could not be controlled, 8 patients gained vision of PL, 3 patients gained vision of 6/ 60-1/ 60 [**9**].

Another case of EE with liver abscess, reported by Wong et al. in 2007, was treated with intravenous cefuroxime and intravitreal vancomycin and amikacin. The patient gained a vision of 6/ 12 [**10**]. 

In 2011, Ishii et al. reported an EE case associated with liver abscess and *Klebsiella pneumoniae* septicemia. The patient was treated timely with pars plana vitrectomy (PPV)+ Lensectomy+ Silicon fitted intraocular lens (SFIOL) and regained vision of 6/ 6 [**11**].

In 2011, Dehghani et al. reported a case of EE associated with liver abscess, treated with intravitreal ceftazidime and vancomycin & PPV & systemic ciprofloxacin. The patient recovered vision of light perception only [**12**].

In 2015, Tsai et al. reported a diabetic patient with liver abscess subsequently developing EE and subdural abscess because of septicemia. The patient was treated with intravenous antibiotics, pars plana vitrectomy, as well as intravitreal ceftazidime and amikacin. The patient recovered vision of 6/ 6 [**13**]. 

Another bilateral EE case was reported by Moore et al. in 2015 and associated liver abscess treated with systemic and intravitreal antibiotics, oral, topical and intravitreal steroids and ultimately PPV, pt. gained good vision of 6/ 12 in R/ E and 6/ 24 in L/ E by this intensive treatment regimen [**14**].

Recently, in 2018, Kim et al. reported a case of EE associated with liver abscess, which was treated with intravenous cefotaxime, metronidazole and amikacin along with pars plana vitrectomy, but could not recover any vision (no light perception) [**15**].

In 2018, Wu MY et al. reported a case of B/ L EE associated with liver abscess, UTI, pneumonia, which was treated with intravenous ceftriaxone. The patient regained vision of 6/ 60 B/ E [**16**].

In all these case reports, laboratory reports revealed that the patients had *Klebsiella pneumoniae* septicemia. Therefore, current evidence, though anecdotal, revealed that *Klebsiella * septicemia is the most important cause of EE in liver abscess patients and can be treated effectively with intravenous 3rd generation cephalosporins. If severe intraocular infection is present, then intravitreal antibiotics and pars plana vitrectomy should also be considered at the earliest in order to preserve vision (**Table 1**, **2**). In 2003, Yoon et al. concluded that *Klebsiella pneumoniae* EE incidence is increasing and if managed aggressively with early PPV and intravitreal injections, could lead to better visual outcomes as compared to conservative treatment that can increase chances of evisceration and enucleation. Early PPV decreases the bacterial and inflammatory load and enhances the antibiotics penetration [**17**].

**Table 1:** Summary of different studies with age, gender and eye affected

Sr. No.	Author, Journal, Year of study	Age	Sex	Eye affected
1.	Dogra et al., IJO 2019 [**2**]	35	M	B/ L
2.	Kim et al., CMH 2018 [**15**]	55	F	R/ E
3.	Rubin et al., CAN J Ophthalmol 2018 [**31**]	68	M	L/ E
4.	Wu et al., Reports 2018 [**16**]	64	M	B/ L
5.	Xu H et al., BMC Ophthalmol 2018 [**34**]	61	M	R/ E
6.	Mali et al., JAMA Ophthalmol 2015 [**39**]	50	F	L/ E
7.	Tsai et al., BMC Ophthalmology 2015 [**13**]	56	M	L/ E
8.	Moore et al., MJA 2015 [**14**]	51	M	B/ L
9.	Tan et al., Eye 2014 [**44**]	78	F	R/ E
10.	Sahu et al., Int Ophthalmol 2013 [**36**]	22-30	F	3 L/ E, 1 R/ E
11.	Malathi et al., case reports in Ophthalmol. Med 2012 [**32**]	18	M	R/ E
12.	Carcasi et al., Nefrologia 2012 [**26**]	51, 78	M, F	L/ E, L/ E
13.	Dehgani et al., Case Report Ophthalmol 2011 [**12**]	79	M	L/ E
14.	Rahman et al., Int. Ophthalmol 2011 [**35**]	26	F	R/ E
15.	Wu et al., CAN J Ophthalmol 2011 [**33**]			
16.	Whist et al., Ophthalmology & Eye diseases 2011 [**41**]	45	F	R/ E
17.	Chheda et al., ARCH Ophthalmol 2011 [**38**]	54	M	L/ E
18.	Ishii et al., Int Ophthalmol 2011 [**11**]	80	F	L/ E
19.	Itoh et al., Case report Ophthalmol 2010 [**24**]	56.5	M	1 R/ E, 1 L/ E
20.	Ang et al., Eye 2010 [**42**]	55	F	B/ L
21.	Hayasaka K et al., Int Ophthalmol 2008 [**27**]	76	M	R/ E
22.	Yodprom et al., Ocular immunology and inflammation 2007 [**30**]	54	M	L/ E
23.	Yang et al., Ophthalmology 2007 [**9**]	33-78	17 M, 5F	B/ L in 5
24.	Wong et al., HKMJ 2007 [**10**]	49	M	R/ E
25.	Saleem et al., NDT 2007 [**25**]	75	M	L/ E
26.	Dua S et al., Am J Transplant 2006 [**22**]	28	F	B/ E L>R
27.	Motley et al., Retina 2005 [**23**]	25	M	L/ E
28.	Chan et al., Am. J. Ophthalmol 2005 [**21**]	69	F	B/ L sequential
29.	Subramanian et al., ARCH Ophthalmol 2003 [**37**]	48	F	R/ E
30.	Tang et al., The Lancet 2003 [**8**]	60	M	L/ E
31.	Arcieri et al., BJID 2001 [**3**]	49	M	B/ L
32.	Betriu et al., JCM 2001 [**29**]	62	M	L/ E
33.	Menon et al., Eye 2000 [**43**]	57	M	R/ E
34.	Reedy et al., Intensive care med 2000 [**28**]	71	F	L/ E
35.	Cahil et al., Br J Ophthalmol 2000 [**6**]	40	M	B/ E
36.	Arroyo, ANN Ophthalmol 2000 [**40**]	57	M	B/ L
37.	Ang et al., Eye 2000 [**7**]	37-85	2M,2F	2L/ E, 1R/ E, 1B/ L

**Table 2:** Summary of endogenous endophthalmitis case reports

Sr. No.	Author, Journal, year of study	No. of cases	Underlying infection	Organism causing EE	Drug sensitivity	Final visual outcome
1.	Dogra, IJO 2019 [**2**]	1	Pancreatic pseudocyst	Klebsiella pneumoniae	intravitreal vancomycin + ceftazidime + colistin, Intravenous colistin, Topical steroids and cycloplegics	OD 6/ 6 OS 6/ 9
2.	Kim et al., CMH 2018 [**15**]	1	Liver abscess	Klebsiella pneumoniae	Intravenous cefotaxime, metronidazole and amikacin, Vitrectomy	NOPL
3.	Rubin et al., CAN J Ophthalmol 2018 [**31**]	1	Infected gall bladder in a diabetic CKD pt.	Klebsiella pneumoniae	intravitreal vancomycin + dexamethasone + amikacin, PPV, Intravenous ceftriaxone and oral moxifloxacin	PL
4.	Wu et al., Reports 2018 [**16**]	1	Liver abscess, pneumonia, UTI in a diabetic pt.	Klebsiella pneumoniae	Intravenous ceftriaxone	6/ 60 OU
5.	Xu et al., BMC [**34**] Ophthalmol 2018	1	Endoscopy in peptic ulcer pt.	Klebsiella pneumoniae	intravitreal ceftazidime, PPV, Retinotomy and abscess aspiration	HM
6.	Mali et al., JAMA Ophthalmol 2015 [**39**]	1	Dental cleaning	Streptococcus intermedius	intravitreal vancomycin + Clindamycin, systemic antibiotics	20/ 25
7.	Tsai et al., BMC Ophthalmology 2015 [**13**]	1	DM, Liver abscess, subdural abscess	Klebsiella pneumoniae	PPV + intravitreal ceftazidime n amikacin	6/ 6
8.	Moore et al., MJA 2015 [**14**]	1	Liver	Klebsiella pneumoniae	Systemic ceftriaxone, oral and topical steroids, intravitreal vancomycin + ceftazidime + dexamethasone, PPV	6/ 12 OD, 6/ 24 OS
9.	Tan et al., Eye 2014 [**44**]	1	Phlebitis	Serratia marcescens	Intravenous ceftriaxone + vancomycin then switched to Meropenem, Daptomycin, Doxycycline, Topical antibiotics and antiglaucoma, Evisceration	NOPL
10.	Sahu et al., Int Ophthalmol 2013 [**36**]	4	Pregnancy, abortion	Bacillus mycoides (1), Klebsiella pneumoniae (1), None (2)	1. Systemic, topical, intravitreal ceftazidime, vancomycin and dexamethasone, 2. oral and topical ofloxacin, 3. PPV, 4. Oral itraconazole	CF to NOPL
11.	Malathi et al., case reports in Ophthalmol. Med. 2012 [**32**]	1	Diarrhoea for 10 days	Salmonella typhi. + fungus	Systemic antibiotics, intravitreal Amphotericin B+ Vanco + ceftazidime	
12.	Carcasi et al., Nefrologia 2012 [**26**]	2	Tunneled haemodialysis catheter	Staph. aureus	Systemic Vancomycin and gentamycin, vitrectomy and intravitreal vancomycin and ceftazidime	NOPL
13.	Dehgani et al., Case Report Ophthalmol 2011 [**12**]	1	Liver abscess	Klebsiella pneumoniae	intravitreal vancomycin + ceftazidime, PPV, systemic Ciprofloxacin	PL
14.	Rahman et al., Int. Ophthalmol 2011 [**35**]	1	PROM	Sphingomonas paucimobilis	intravitreal vancomycin + amikacin, Oral Moxifloxacin and steroids	6/ 9
15.	Wu et al., CAN J Ophthalmol 2011 [**33**]	1	Colonoscopy	E. coli	intravitreal vancomycin + amikacin + ceftazidime, PPV & lensectomy, Intravenous vancomycin + Metronidazole + ciprofloxacin	NOPL
16.	Whist et al., Ophthalmology & Eye diseases 2011 [**41**]	1	Systemically well	Staph epidermidis	intravitreal vancomycin + amikacin + foscarnet, PPV and lensectomy, Intravenous vancomycin	HM
17.	Chheda et al., ARCH Ophthalmol 2011 [**38**]	1	Brain abscess after tooth extraction	S. constellatus	intravitreal vancomycin + Clindamycin + ceftazidime, Intravenous ceftriaxone + Metronidazole	6/ 60
18.	Ishii et al., Int Ophthalmol 2011 [**11**]	1	Liver abscess	Klebsiella pneumoniae	PPV + Lensectomy + SFIOL	6/ 6
19.	Itoh et al., Case report Ophthalmol 2010 [**24**]	2	1. After heart surgery- endocarditis, gingivitis, brain abscess, 2. Acute pyelitis and prostatic abscess	1. Streptococcus anginosus, 2. Staphylococcus sp	1. PPV + systemic imipenem, 2. intravitreal ceftazidime + vancomycin + systemic imipenem	1. 6/ 7.5, 2. 6/ 4.8
20.	Ang et al., Eye 2010 [**42**]	1	Systemically well	P. acne	Intravenous crystalline penicillin, topical moxifloxacin, prednisolone, Oral steroids	6/ 9 B/ L
21.	Hayasaka et al., Int Ophthalmol 2008 [**27**]	1	Liver cancer, pulm. T.B., Spondylitis	Streptococcus bovis	PPV + SOI, Intravenous Meropenem	6/ 60
22.	Yodprom et al., Ocular immunology and inflammation 2007 [**30**]	1	HIV	Salmonella choleraesuis	Intravitreal vancomycin + ceftazidime, Intravenous ceftriaxone	NOPL
23.	Yang et al., Ophthalmology 2007 [**9**]	22	Liver abscess, DM in 15 patients, biliary stones in 2	Klebsiella pneumoniae	Systemic antibiotics, 3rd generation cephalosporins and aminoglycosides	NOPL in 11 (evisceration), PL in 8, 6/ 60-1/ 60 in 3
24.	Wong et al., HKMJ 2007 [**10**]	1	liver abscess	Klebsiella pneumoniae	Intravenous cefuroxime + intravitreal vancomycin + amikacin	6/ 12
25.	Saleem et al., NDT 2007 [**25**]	1	Dialysis catheter exit site infection related septicemia	Staph. aureus	Intravenous Flucloxacillin + intravitreal vancomycin and amikacin	6/ 12
26.	Dua et al., Am J Transplant 2006 [**22**]	1	B/ L lung transplantation for end stage bronchiectasis secondary to CF	Pseudomonas aeruginosa	Intravitreal cefta + vanco + amphotericin B, Systemic vancomycin, piperacillin, tazobactam, colistin, PPV L/ E	HM
27.	Motley et al., Retina 2005 [**23**]	1	Cystic fibrosis	Pseudomonas aeruginosa	Systemic ceftazidime + tobramycin + ciprofloxacin, intravitreal and subconj. Antibiotics, enucleation	NOPL
28.	Chan et al., Am. J. Ophthalmol 2005 [**21**]	1	Bronchiectasis	Pseudomonas aeruginosa	Systemic and intravitreal ceftazidime, PPV	20/ 40
29.	Subramanian et al., ARCH Ophthalmol 2003 [**37**]	1	Dental cleaning	α-hemolytic streptococci	intravitreal vancomycin + amikacin, PPV	CF at 1 m
30.	Tang et al., The Lancet 2003 [**8**]	1	Suppurative liver ds, DM	Klebsiella pneumoniae	Cefotaxime, Intravitreal vancomycin + amikacin	NOPL
31.	Arcieri et al., BJID 2001 [**3**]	1	Infective endocarditis	Gram positive coccobacillus group B Streptococcus	Fluoroquinolones	PL OS, NOPL OD
32.	Betriu et al., JCM 2001 [**29**]	1	CA. Larynx, Laryngectomy done, on radiation therapy and steroids	Listeria monocytogenes	Oral ciprofloxacin plus topical fort. Vancomycin and intravitreal vancomycin	HM
33.	Menon et al., Eye 2000 [**43**]	1	Not found	Pseudomonas aeruginosa	Systemic cefotaxime and steroids, intravitreal vancomycin + amikacin	NOPL
34.	Reedy et al., Intensive care med 2000 [**28**]	1	Cholangiocarcinoma complicated by ascending cholangitis	Pseudomonas aeruginosa	Topical Cefazoline + tobramycin Intravitreal vancomycin + tobramycin, Oral ciprofloxacin	NOPL
35.	Cahil et al., Br J Ophthalmol 2000 [**6**]	1	Liver abscess	Klebsiella pneumoniae	Intravenous Ciprofloxacin 400mg twice daily and hydrocortisone 100 mg three times, Topical antibiotic, steroid and mydriatic, PPV + retinopexy	PL in R/ E, 6/ 12 in L/ E
36.	Arroyo, Ann Ophthalmol 2000 [**40**]	1	Prostate abscess	Staph. aureus	intravitreal vancomycin + ceftazidime + amikacin, PPV, topical and systemic antibiotics	OD 6/ 6, OS PL +
37.	Ang et al., Eye 2000 [**7**]	4	Pneumonia in 2 pt, Liver abscess and UTI in 1 pt each	Klebsiella pneumoniae	Intravitreal, S/ C, Topical cefazoline and gentamycin, Intravenous ceftriaxone + gentamycin	NOPL in 3 pts, 6/ 6 in 1 patient

In 2014, Sridhar et al. reported that endogenous *Klebsiella* pneumoniae endophthalmitis (EKPE) is associated with poorer visual outcomes and higher rates of evisceration and enucleation as compared to exogenous *Klebsiella pneumoniae* endophthalmitis [**18**].

In 2016, Odouard et al. reported that time since presentation from the onset of symptoms is crucial, as late presentation can increase chances of evisceration and enucleation. In addition, this early PPV and intravitreal antibiotic and corticosteroid injections can lead to a better visual outcome [**19**].

In 2017, Shields et al. reported that EKPE is associated with poor visual outcomes, 58% of the eyes in their series had a final visual outcome of LP or NLP. EKPE is commonly seen in patients of Asian ethnicity with liver abscess. Early detection and aggressive treatment can lead to better visual outcome [**20**].

**Pulmonary diseases**

In 2000, Ang et al. reported 2 cases of EE associated with pneumonia and *Klebsiella pneumoniae* septicemia, treated with intravitreal, topical, subconjunctival cefazoline and gentamycin and intravenous ceftriaxone and gentamycin [**7**]. One patient could not regain any vision and one patient gained vision of 6/ 6 B/ E. The difference in visual outcome was explained by the time lapse in presentation from the onset of symptoms. The patient with NOPL visual outcome presented later than the patient who gained vision of 6/ 6 (**Table 1**, **2**).

In 2005, Chan et al. reported a case of EE associated with bronchiectasis. The patient was treated with systemic and intravitreal ceftazidime and PPV. The patient attained good vision of 20/ 40 [**21**].

In 2006, Dua et al. reported a case of EE in a patient with B/ L lung transplantation for end stage bronchiectasis secondary to CF. The patient was treated with Intravitreal cefta + vanco + amphotericin B and systemic vancomycin, piperacillin, tazobactam, colistin and PPV, but could gain vision of HM only [**22**].

In 2015, Motley et al. reported a case of EE and choroidal abscess associated with cystic fibrosis. The patient was treated with intravenous ceftazidime, ciprofloxacin and tobramycin, intravitreal and subconjunctival injections of same antibiotics, retinectomy and abscess excision, but the intraocular infection could not be controlled and ultimately the patient required enucleation [**23**].

In all these three pulmonary diseases associated cases of EE, the causative organism was *Pseudomonas aeruginosa*. 

**Infective endocarditis**

In 2001, Arcieri et al. reported a patient who developed bilateral EE following *group B Streptococcus* septicemia along with infective endocarditis. The patient was treated with intravenous fluoroquinolones, but could only recover perception of light in one eye, while the other eye could not perceive light [**3**]. 

In 2010, Itoh et al reported a case of EE in a patient after heart surgery. After surgery, the patient developed septicemia, endocarditis, gingivitis and brain abscess. *Streptococcus anginosus* was the causative agent. The patient was treated with PPV and systemic imipenem. The patient achieved good vision of 6/ 7.5 [**24**]. 

While the evidence is limited, *gram positive streptococci* septicemia in infective endocarditis patients is the most commonly reported cause of EE. This infection may be amenable to treatment with intravenous penicillin and fluoroquinolones. However, visual results reported so far are not encouraging with most patients requiring surgical interventions like enucleation or pars plana vitrectomy (**Table 1**, **2**).

**Tunnelled haemodialysis catheters**

In 2007, Saleem et al. reported a case of EE associated with a dialysis catheter exit site infection and *Staphylococcus aureus* blood stream infection (BSI). This patient was treated with intravenous flucloxacillin and intravitreal vancomycin and amikacin, and recovered a vision of 6/ 12 [**25**].

In 2012, Carcasi et al. also reported a similar case of EE associated with dialysis catheter exit site infection and *Staphylococcus aureus* blood stream infection. The patient was treated with intravenous vancomycin and gentamycin along with intravitreal vancomycin and ceftazidime. Despite pars plana vitrectomy, the patient could not recover any vision (No PL) [**26**].

Thus, *Staphylococcus aureus* has been the most common bacterium reported causing EE in patients having dialysis catheter associated BSI. These patients may be treated with i.v. vancomycin and third generation cephalosporins and intravitreal antibiotics. Fulminant intraocular infection has a relatively poor prognosis and the patient may not recover useful vision even after pars plana vitrectomy (**Table 1**, **2**).

**Immunosuppression**

In 2000, Hayasaka et al. reported a case of EE in a liver cancer and pulmonary T.B. patient suffering from *Streptococcus bovis* bacteremia. The patient received treatment with vitrectomy and SOI and intravenous meropenem, but could only gain vision of 6/ 60 [**27**].

In the same year, Reedy et al. reported a case of EE associated with Cholangiocarcinoma and *Pseudomonas aeruginosa* septicemia. The patient was treated with topical Cefazoline + tobramycin, Intravitreal vancomycin + tobramycin and oral ciprofloxacin, but the patient’s visual outcome was NO PL [**28**].

In 2001, Betriu et al. reported a case of *Listeria monocytogenes* EE in a patient with cancer of the larynx, who was undergoing radiotherapy and was on steroids. The patient was administered oral ciprofloxacin and intravitreal vancomycin, but the vision recovery was only hand movements close to face [**29**]. 

In 2007, Yodoprom et al. reported a case of *Salmonella choleraesuis* EE in a HIV infected individual. The patient was treated with intravitreal vancomycin, ceftazidime and intravenous ceftriaxone. But the patient’s visual outcome was NO PL [**30**].

In 2018, Rubin et al. reported a case of *Klebsiella pneumoniae* EE associated with infected gall bladder in a diabetic CKD patient. The patient was treated with intravitreal vancomycin, dexamethasone, ceftazidime and intravenous ceftriaxone, oral Moxifloxacin and PPV. But the patient could only gain vision of PL [**31**].

**Diarrhoeal disease**

In 2012, Malathi et al. reported a case of EE in a patient having diarrhea for 10 days. Blood culture of the patient yielded Salmonella typhi and fungus, the patient being treated with systemic antibiotics and intravitreal Amphotericin B, vancomycin and ceftazidime, but the eye could not be salvaged and ultimately required evisceration [**32**] (**Table 1**, **2**).

**Invasive diagnostic procedures**

In 2011, Wu et al. reported a case of EE associated with post colonoscopy bacteremia with *E. coli*. The patient was treated with intravitreal vancomycin and ceftazidime, and intravenous vancomycin, metronidazole and ciprofloxacin and PPV. But the patient’s visual outcome was NO PL [**33**].

In 2018, Xu et al. reported a case of *Klebsiella pneumoniae* EE after endoscopy for peptic ulcer in a diabetic heavy drinker with history of recent significant weight loss. The patient was treated with intravitreal ceftazidime, PPV, retinotomy and retinal abscess drainage. But the patient’s visual outcome was only HM [**34**].

**Pregnancy**

In 2011, Rahman et al. reported a case of *Sphingomonas paucimobilis* EE in a post-partum lady with PROM. The patient was treated with intravitreal vancomycin and amikacin, oral moxifloxacin and steroids. The patient gained vision of 6/ 9 [35] (**Table 1**, **2**).

In 2013, Sahu et al. reported 4 cases of EE associated with pregnancy and abortion. In 1 patient the causative organism was *Bacillus mycoides*, in another patient *Klebsiella pneumoniae*, and in 2 patients no organism was identified. The patients were treated with systemic, topical, intravitreal ceftazidime, vancomycin and dexamethasone, oral and topical ofloxacin, PPV and oral itraconazole but in all the 4 patients the visual outcome was very poor (NOPL to CF) [**36**].

**Dental procedures**

In 2003, Subramanian et al. reported a case of *α hemolytic streptococci* EE after dental cleaning. The patient was treated with intravitreal vancomycin and amikacin and PPV, but the patient could not gain vision of counting finger at only 1 m [**37**]. 

In 2011, Chheda et al. reported a case of EE after tooth extraction. *Streptococcus constellatus* bacteremia caused brain abscess and EE in this patient. The patient was treated with intravitreal vancomycin, ceftazidime, clindamycin and intravenous ceftriaxone, metronidazole but the patient could gain vision of 6/ 60 [**38**].

Another case of EE after dental cleaning was reported by Mali JO et al. in 2015, [**39**] the patients investigations revealed *Streptococcus intermedius* as the causative agent. The patient was treated with intravitreal vancomycin and clindamycin and systemic antibiotics. The patient regained vision of 20/ 25 (**Table 1**, **2**)

**Pancreatic pseudocyst**

In 2019, Dogra M et al. reported a case of *Klebsiella pneumoniae* B/ L EE, in a patient with pancreatic pseudocyst. The patient was treated with intravitreal vancomycin, ceftazidime, topical steroids and cycloplegics, intravenous and intravitreal colistin. The patient gained good vision of 6/ 6 in R/ E and 6/ 9 in L/ E [2] (**Table 1**, **2**).

**Prostate abscess**

In 2000, Arroyo reported a case of EE associated with *Staphylococcus sp* septicemia and prostate abscess. The patient was treated with intravitreal vancomycin + ceftazidime + amikacin, PPV, topical and systemic antibiotics. The patient gained vision of 6/ 6 OD, PL+ OS [**40**].

In 2010, Itoh et al reported a similar case treated with intravitreal ceftazidime + vancomycin and systemic imipenem. The patient’s visual outcome was 6/ 4.8 [**24**].

**Systemically well patient**

In 2011, Whist et al. reported a case of staph epidermidis EE in a systemically well patient. The patient was treated with intravitreal foscarnet + vancomycin + amikacin, intravenous vancomycin, PPV and lensectomy. The patient regained vision of HM [**41**] (**Table 1**, **2**).

In 2010, Ang et al. reported a case of *Propionibacterium acne* B/ L EE in a systemically well patient. The patient was treated with topical moxifloxacin + prednisolone and intravenous crystalline penicillin and oral steroids. The patient gained good vision of 6/ 9 in B/ E [**42**]. Another case of EE reported by Menon et al. in 2000 [**43**] associated with *P. aeruginosa* septicemia, in which the patient was treated with systemic cefotaxime and steroids and intravitreal injections of vancomycin and amikacin, but the patient could not recover any vision. So, it is obvious that *P. aeruginosa* septicemia associated EE generally has a poor visual prognosis despite intensive medical and surgical treatment.

**Phlebitis**

In 2014, Tan et al. reported a case of *Serratia marcescens* EE in a patient with phlebitis after intravenous cannulation. The patient was treated with intravenous ceftazidime + vancomycin, topical antibiotic and antiglaucoma drugs. The patient was then switched to meropenem, then to daptomycin and doxycycline but the ocular inflammation could not be controlled, ultimately the patient requiring evisceration [**44**] (**Table 1**, **2**).

## Conclusion

While the evidence for the associations of endogenous endophthalmitis is extremely limited, it is obvious that the most common site of primary infection for EE is the liver (liver abscess). Other primary foci include lungs (pneumonia, CF, bronchiectasis), heart (infective endocarditis), tunneled hemodialysis catheter exit site infection, and meningitis [**45**-**48**].

Even though endogenous endophthalmitis is a rare entity nowadays, especially because of the availability of effective antimicrobial agents, it must be kept in mind in immunocompromised patients. Diabetics, cancer patients on immunosuppression, patients on steroids, hospitalized patients with intravenous access, and patients with renal diseases on dialysis are especially vulnerable to metastatic endophthalmitis. Systemic antibiotic treatment and systemic antifungal treatment (the latter, in case of fungal EE and fungal septicemia) is usually sufficient to control the EE along with the primary site of infection. Choice of antibiotic depends upon culture and sensitivity reports of blood, urine, CSF, and local wound swabs [**49**-**52**]. 

In cases with fulminant intraocular inflammation and infection, aqueous and vitreous aspirates culture and sensitivity may guide the choice of intravitreal antibiotics. If the infection is not controlled even with this, then pars plana vitrectomy should be considered at the earliest in order to decrease the infectious agent and toxin load. Even after this, if the infection is not controlled then enucleation or rarely, evisceration, may be performed [**53**-**58**]. 

**Conflict of Interest**

There is no conflict of interest between authors.

**Funding**

No funding was taken to conduct the study.
